# Inhibition of SP1 by the mithramycin analog EC-8042 efficiently targets tumor initiating cells in sarcoma

**DOI:** 10.18632/oncotarget.8817

**Published:** 2016-04-18

**Authors:** Juan Tornin, Lucia Martinez-Cruzado, Laura Santos, Aida Rodriguez, Luz-Elena Núñez, Patricia Oro, Maria Ana Hermosilla, Eva Allonca, Maria Teresa Fernández-García, Aurora Astudillo, Carlos Suarez, Francisco Morís, Rene Rodriguez

**Affiliations:** ^1^ Hospital Universitario Central de Asturias and Instituto Universitario de Oncología del Principado de Asturias, Oviedo, Spain; ^2^ EntreChem SL, Oviedo, Spain; ^3^ Unidad Histopatología Molecular en Modelos Animales de Cáncer, IUOPA, Universidad de Oviedo, Oviedo, Spain; ^4^ Servicio de Anatomía Patológica, Hospital Universitario Central de Asturias, Oviedo, Spain

**Keywords:** myxoid liposarcoma, mesenchymal stem cells, cancer stem cells, mithralog EC-8042, DIG-MSK

## Abstract

Tumor initiating cells (TICs), responsible for tumor initiation, and cancer stem cells (CSCs), responsible for tumor expansion and propagation, are often resistant to chemotherapeutic agents. To find therapeutic targets against sarcoma initiating and propagating cells we used models of myxoid liposarcoma (MLS) and undifferentiated pleomorphic sarcoma (UPS) developed from human mesenchymal stromal/stem cells (hMSCs), which constitute the most likely cell-of-origin for sarcoma. We found that SP1-mediated transcription was among the most significantly altered signaling. To inhibit SP1 activity, we used EC-8042, a mithramycin (MTM) analog (mithralog) with enhanced anti-tumor activity and highly improved safety. EC-8042 inhibited the growth of TIC cultures, induced cell cycle arrest and apoptosis and upregulated the adipogenic factor CEBPα. SP1 knockdown was able to mimic the anti-proliferative effects induced by EC-8042. Importantly, EC-8042 was not recognized as a substrate by several ABC efflux pumps involved in drug resistance, and, opposite to the chemotherapeutic drug doxorubicin, repressed the expression of many genes responsible for the TIC/CSC phenotype, including *SOX2*, *C-MYC*, *NOTCH1* and *NFκB1*. Accordingly, EC-8042, but not doxorubicin, efficiently reduced the survival of CSC-enriched tumorsphere sarcoma cultures. *In vivo*, EC-8042 induced a profound inhibition of tumor growth associated to a strong reduction of the mitotic index and the induction of adipogenic differentiation and senescence. Finally, EC-8042 reduced the ability of tumor cells to reinitiate tumor growth. These data suggest that EC-8042 could constitute an effective treatment against both TIC and CSC subpopulations in sarcoma.

## INTRODUCTION

Sarcomas often show a limited clinical response to cytotoxic drugs which still remain as the most utilized agents for generalized treatment of soft tissue sarcomas [1]. A hypothesis to explain the resistance of sarcomas to chemotherapy is the existence of drug-resistant CSCs, which are responsible of tumor progression and propagation. These subpopulations of CSCs emerge during tumor evolution from the cell-of-origin or TICs, which are the normal cells that acquire the first cancer-promoting mutations and initiate tumor formation [2]. It has been recently established that transformed MSCs and/or their immediate lineage progenitors are the most likely cell-of-origin for many types of sarcomas [3, 4]. Accordingly, many efforts have been undertaken to produce models of sarcomas based on MSCs transformed with relevant oncogenic events. This kind of models constitutes unparalleled systems to unravel the mechanisms underlying sarcomagenesis from the cell of origin, to explore the evolution of CSCs subpopulations and to search for specific therapies able to eliminate these populations.

In the search for new therapeutic targets, transcription factors (TFs) presenting altered activity offer a promising choice since they are pivotal points in signaling pathways and therefore their inhibition may block several routes involved in tumor progression [5]. In this line, several members of the SP family of TFs, such as SP1, SP3 and SP4, have been found to play an important role in the pathogenesis of some cancers including sarcomas and may constitute relevant therapeutic targets [6–10]. MTM is an antitumoral antibiotic natural product that binds preferentially to GC-rich sequences in DNA and prevents the binding of SP TFs in gene promoters, thus inhibiting the transcription of SP-regulated genes [11]. MTM showed strong response rates in cancer treatment although its clinical use was limited due to its adverse side effects [12]. Nevertheless, new studies reporting specific modes of action in certain histologies, like Ewing sarcoma [13], has renewed the interest in MTM and have warranted the development of two clinical trials (http://www.clinicaltrials.gov, NCT016110570 and NCT01624090).

Genetic engineering of the MTM biosynthesis pathway has been used to generate mithralogs showing an efficient inhibition of the SP-mediated gene expression and bearing both lower toxicity and higher biological activity [14–16]. Recently, the new mithralog demycarosyl-3D-β-D-digitoxosyl-mithramycin SK (EC-8042; DIG-MSK) has been obtained and characterized [17]. As expected, EC-8042 preferentially binds GC-rich sequences, is a potent inhibitor of SP-driven gene expression and shows pleiotropic anti-angiogenic and anti-oncogenic activities [18–20]. EC-8042 altered the expression of cell cycle related genes resulting in cell cycle arrest and apoptosis in breast cancer cell lines [21]. In Ewing sarcoma, EC-8042 was substantially less toxic than mithramycin but maintained suppression of EWS-FLI1 at similar concentrations as MTM, and markedly suppressed Ewing sarcoma xenograft growth [22]. More importantly, EC-8042 shows a potent *in vitro* and *in vivo* antitumor activity against several tumors types similar to other analogues but is 10-fold less toxic *in vivo* than MTM [17], enabling it as the lead candidate in the quest for mithralogs with improved therapeutic window potentially useful in the clinical setting.

We have recently established and characterized pioneer models of UPS and MLS developed from sequentially mutated human bone marrow-derived MSCs (hBMSCs) [23–25]. To find specific therapies able to target TICs in sarcoma we search for altered TF-mediated signaling in these sarcoma models. We found that SP1-mediated transcription was among the most significantly altered signaling and that the inhibition of SP1 expression and activity by EC-8042 efficiently inhibited the *in vitro* and *in vivo* growth in these models of sarcoma initiating cells. EC-8042 repressed the expression of important genes associated to the TIC/CSC phenotype and was able to inhibit the growth of tumorsphere cultures and the ability to reinitiate tumor growth, suggesting that EC-8042 could constitute an effective treatment against both the TIC and the CSC subpopulations in sarcoma.

## RESULTS

### SP1-mediated signaling is altered in sarcoma-initiating transformed hBMSCs

We have previously developed and characterized sarcoma models using hBMSCs sequentially mutated with up to 5 oncogenic events. This collection of hBMSCs ranges from wt (MSC-0H) to fully transformed hBMSCs (MSC-5H). In addition, the fusion oncogene FUS-CHOP (FC), characteristic of MLS, or the corresponding GFP-control, is ectopically expressed in all the MSC types (Supplementary Figure S1A) [23–25]. MSC-4H-GFP cells are immortalized, but not transformed and the rest of the MSC types are transformed and originate sarcomas *in vivo* [23, 25]. Thus, MSC-5H-GFP give rise to pleomorphic undifferentiated sarcomas, meanwhile FUS-CHOP-expressing hBMSCs (MSC-4H-FC, and MSC-5H-FC) initiate MLS-like tumors. Several cell lines were also derived from xenograft tumors generated by transformed hMSCs (T-4H-FC#1, T-4H-FC#3, T5H-GFP#1 and T5H-FC#1) (Supplementary Figure S1A).

**Figure 1:**
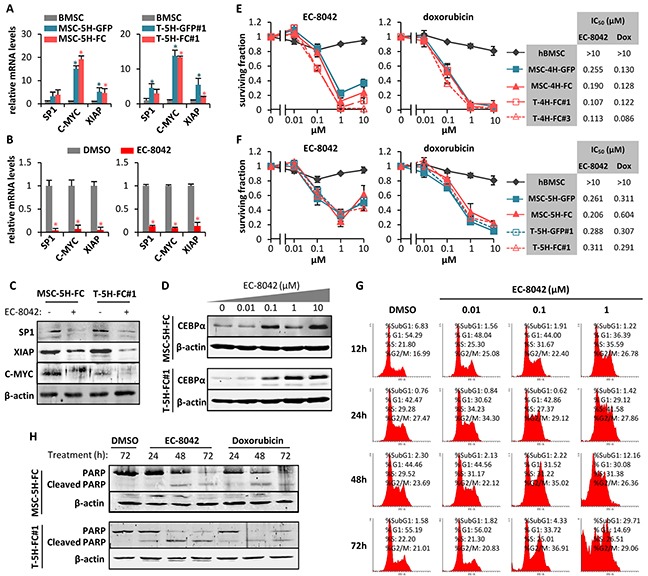
Antiproliferative effects of EC-8042 **A.** mRNA expression of SP1 and SP1-downstream genes (C-MYC and XIAP) in MSC5H-GFP and MSC-5H-FC (left panel) and T-5H-GFP#1 and T-5H-FC#1 cells (right panel) relative to hBMSCs. **B.** mRNA expression of SP1 and SP1-downstream genes (C-MYC and XIAP) in MSC5H-FC (left panel) and T5H-5H-FC#1 cells (right panel) treated with 0.5μM EC-8042 for 24 hours, relative to untreated (DMSO) controls. **C.** Protein levels of SP1, C-MYC, XIAP and β-actin in MSC5H-FC and T5H-5H-FC#1 cells treated with 0.5μM EC-8042 for 24 hours. **D.** C/EBPα protein level upregulation after 48h- (MSC-5H-FC) or 24h-treatment (T-5H-FC#1) with the indicated concentrations of EC-8042. **E–F.** Cell viability (WST1 assay) measured after the treatment of wild-type hBMSCs and the indicated MSC-4H and T-4H (E) or MSC-5H and T-5H cell lines (F) with increasing concentrations of EC-8042 (left panels) or doxorubicin (middle panels) for 48 hours. IC_50_ values for each cell type are shown (right panels). **G.** Time-course evolution of the cell cycle distribution of MSC-5H-FC cells treated with increasing concentrations of EC-8042. **H.** Apoptotic cleavage of PARP in MSC-5H-FC and T-5H-FC#1 cells treated with 0.5μM EC-8042 or 0.5μM doxorubicin for the indicated times. The expression of β-actin was used as loading control in western blotting analysis. Error bars represents the standard deviation and asterisks indicate a statistically significant difference with the respective control groups (*:p<0.05; two-sided Student *t* test).

To identify altered signaling in this sarcoma TICs we performed a gene-expression profiling (GEP) comparing wild type and transformed hBMSCs types. Ingenuity Pathway Analysis (IPA) of differentially expressed genes revealed the upstream regulation signaling most significantly altered in MSC-5H-GFP, MSC-5H-FC and T-5H-FC#1 cells (Supplementary Figure S1B). All these cell types displayed a similar pattern of transcription factor-mediated signaling deregulation, with SP1-mediated transcription being the most significantly altered signaling that showed a higher positive activation z-score (a predictive value of activation/inhibition) in all cell lines (Supplementary Figure S1B). Accordingly, RT-PCR analysis showed that the expression of SP1 and two known SP1-target genes, *C-MYC* and *XIAP*, was upregulated in MSC-5H-GFP, MSC-5H-FC, T-5H-GFP#1 and T-5H-FC#1 cells (Figure 1A). Importantly, 24 hours-treatment with 0.5 μM EC-8042 efficiently inhibited the expression of SP1 and their downstream targets, both at mRNA and protein level, in MSC-5H-FC#1 and T-5H-FC#1 cells (Figure 1B–1C). Moreover, EC-8042 also decreased the expression of other members of the SP family which may show certain level of overlap with SP1-induced signaling [6]. Thus, EC-8042 treatment inhibited the expression of SP3 in both MSC-5H-FC#1 and T-5H-FC#1 cell types and SP4 in MSC-5H-FC (Supplementary Figure S2). In addition, it was previously reported that SP1 repressed the expression of the adipogenic factor C/EBPα [26]. In line with this finding, the inhibition of SP1 activity with EC-8042 resulted in a concentration-dependent activation of C/EBPα (Figure 1D).

### Antiproliferative effects of EC-8042

Opposite to wild type hBMSCs, all cell types of the 4H- (Figure 1E) and 5H- models (Figure 1F) were sensitive to sub-micromolar concentrations of EC-8042 or doxorubicin (IC_50_ between 0.107 and 0.311 μM for EC-8042 and between 0.086 and 0.604 μM for doxorubicin). Likewise, EC-8042 showed a similar cytotoxic effect on a low-passaged primary sarcoma patient-derived cell line (Supplementary Figure S3A). To explore the mechanism underlying the antiproliferative effect of EC-8042, we examined the effect of this drug on the cell cycle distribution. MSC-5H-FC cells treated with 0.1 μM EC-8042 showed a slow transition through the S-phase followed by G2 arrest, meanwhile cells treated with a higher concentration also showed a relevant increment in the Sub-G_1_ apoptotic population at latter times (Figure 1G). In line with this result, MSC-5H-FC and T-5H-FC#1 cells treated with low concentrations (0.01-0.05 μM) of EC-8042 stained positive for senescence-associated β-galactosidase activity (Supplementary Figure S4), suggesting the induction of a senescence-like state, while higher concentrations of EC-8042 (0.5 μM) induced a time-dependent poly (ADP-ribose) polymerase (PARP) apoptotic cleavage in a similar way to doxorubicin (Figure 1H). Thus, EC-8042 treatment induced cell cycle arrest followed by apoptotic cell death in sarcoma TICs.

**Figure 2:**
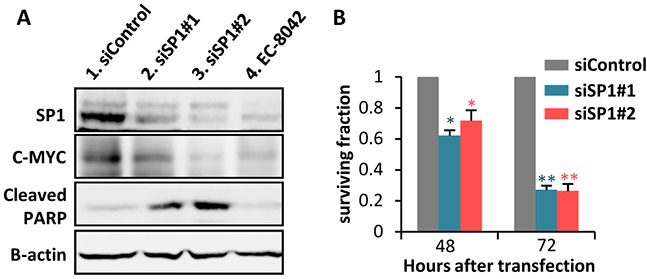
SP1 knockdown mimics EC-8042 antiproliferative effects **A.** Protein levels of SP1, C-MYC and cleaved-PARP in MSC-5H-FC cells 48 hours after the transfection of the indicated siRNAs (lanes 1-3) or after a 24 hours-treatment with 0.5μM EC-8042 (lane 4). β-Actin levels are presented as a loading control. **B.** Effect of siRNAs on the cell viability (WST1 assay relative to siControl values) of MSC-5H-FC cells measured 48 or 72 hours after transfection. Error bars represents the standard deviation (n=3 independent experiments) and asterisks indicate a statistically significant difference with the respective control groups (*:p<0.05, **:p<0.005; two-sided Student *t* test).

To confirm that SP1 inhibition mediates the anti-proliferative effects of EC-8042, we performed siRNA knockdown of this transcription factor in MSC-5H-FC cells. Two different siRNA duplexes inhibit SP1 protein expression to levels comparable to that observed after a 24 hour treatment with EC-8042. These SP1 knockdowns correlate with the inhibition of C-MYC and the induction of PARP apoptotic cleavage (Figure 2A). Likewise, SP1 depletion induces a time-dependent cytotoxic effect (Figure 2B). Therefore, SP1 knockdown seems to mimic the anti-proliferative effects induced by EC-8042.

### EC-8042 decreases the expression of ABC transporters and is not a substrate for them

**Figure 3:**
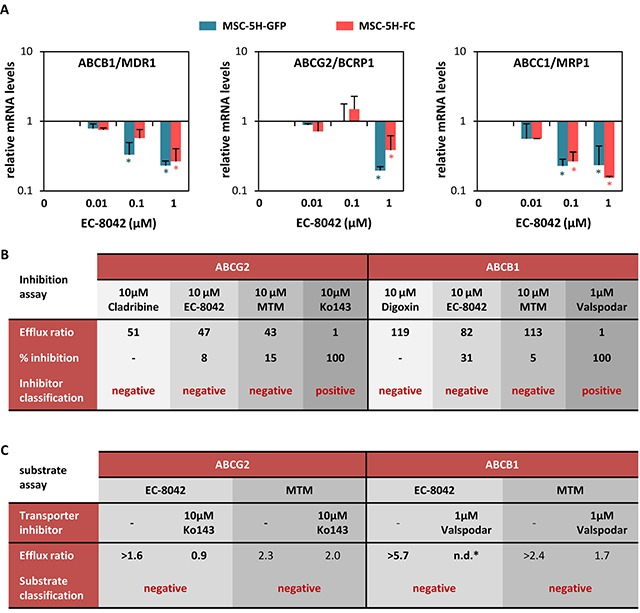
EC-8042 downregulates the expression of ABC transporters and is not a substrate for them **A.** Relative mRNA expression of ABCG2, ABCB1 and ABCC1 genes in MSC-5H-GFP and MSC-5H-FC cells treated with the indicated concentrations of EC-8042 for 24 hours. **B.** ABCG2 and ABCB1 functional inhibition assay for EC-8042 and MTM. Cladribine/Ko243 and digoxin/valspodar were used as negative/positive control drugs for ABCG2 and ABCB1 respectively. **C.** ABCG2 and ABCB1 functional substrate assay for EC-8042 and MTM. Ko243 and valspodar were used as transporter inhibitors for ABCG2 and ABCB1 respectively. Error bars represents the standard error of the mean (SEM) and asterisks indicate a statistically significant difference with the respective control groups (*:p<0.05; two-sided Student *t* test).

Previous reports showed that the expression of several members of the ATP binding cassette (ABC) transmembrane protein family is regulated by SP1 and can be repressed by MTM treatment [27–29]. These ABC proteins act as efflux pumps for drugs and metabolites and cause drug resistance in tumor cells. Therefore, we studied the effect of EC-8042 on the expression of the 3 members of the family most commonly involved in drug resistance of cancer cells. EC-8042 was able to decrease the expression of *ABCB1* (*MDR1*), *ABCG2* (*BCRP1*) and *ABCC1* (*MRP1*) in MSC-5H cells (Figure 3A). Nevertheless, neither MTM nor EC-8042 inhibited the pumping activity of ABC transporters (Figure 3B). More importantly, a functional assay showed that ABCG2 and ABCB1 proteins failed to transport both MTM and EC-8042 (Figure 3C). This behavior is in stark contrast to most chemotherapeutic agents, including doxorubicin, which are known substrates of ABC transporters [30]. Given that drug resistance caused by enhanced ABC proteins expression and activity is a hallmark of both normal stem cells and CSCs [31], the inability of ABC transporters to pump out EC-8042, together with the capacity of this mithralog to repress the expression of ABC transporters genes, suggest that EC-8042 could efficiently target CSC subpopulations.

### EC-8042 decreases the expression of genes associated to the CSC phenotype

**Figure 4:**
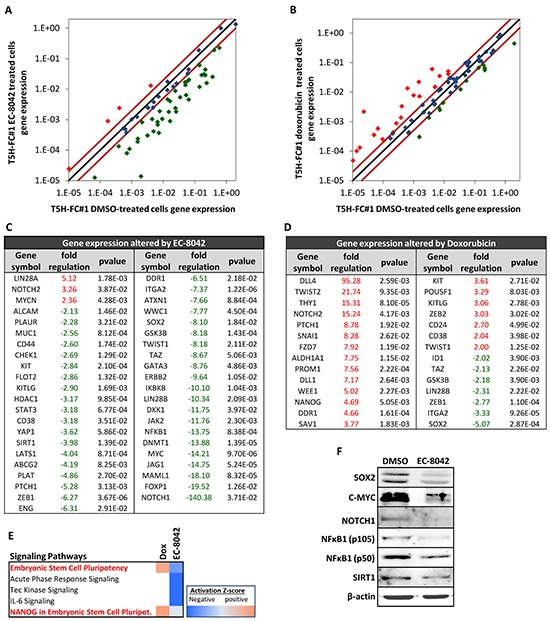
Changes in CSC-related gene expression induced by EC-8042 and doxorubicin RNA derived from T5H-FC#1 cells treated with the carrier substance (DMSO) or with 0.5 μM EC-8042 or 0.5 μM doxorubicin for 24 hours were used to analyzed the expression of 86 CSC-related genes (RT^2^ Profiler™ PCR Array System PAHS-176Z, Qiagen). **A–B.** Scatter plots representing the expression values in control (DMSO) and EC-8042- (A) or doxorubicin- (B) treated cells for each gene. Genes above and below the dark-red lines are expressed more than two fold up (red symbols) or down (green symbols) in treated versus untreated cells respectively. **C–D.** List of genes differentially expressed (fold change ≤-2 or ≥2 and p-value (two sided Student *t* test) < 0.05) after EC-8042 (C) or doxorubicin (D) treatment. **E.** Comparison of genes differentially expressed after EC-8042 and doxorubicin treatment using the IPA software. The heat map of the mostly altered signaling pathways sorted by their variation in the activation z-score is presented. **F.** Expression of SOX2, C-MYC, NOTCH1, NFκB1 (p50 and p105) and SIRT1 proteins in T5H-5H-FC#1 cells treated with 0.5μM EC-8042 for 24 hours. The expression of β-actin was used as loading control.

To gain further insight about the ability of EC-8042 to affect the CSC phenotype in sarcoma, we performed RT-PCR in T-5H-FC#1 cells treated for 24h with 0.5 μM EC-8042 or doxorubicin to analyze the expression of an array of 84 genes with well known functions in pluripotency, self-renewal, migration, metastasis and signal transduction in CSCs as well as several CSC markers. EC-8042 and doxorubicin induced statistical significant changes (fold change≥2; p-value<0.05) in the expression of 42 and 30 genes respectively (Figure 4A–4D). Notably, the way these drugs altered CSC-related gene expression is strikingly different. Most genes affected by doxorubicin treatment were upregulated (21 upregulated genes and 9 downregulated genes) (Figure 4B & 4D), whereas EC-8042-treatment induced the downregulation of almost all the altered genes (3 upregulated genes and 39 downregulated genes) (Figure 4A & 4C). Confirming the effect of EC-8042 over SP1-mediated transcription, several genes known to be controlled by SP1 were downregulated in EC-8042 treated cells, including *HDAC1*, *ITGA2*, *KIT*, *ENG*, *PLAUR* or *C-MYC*. The genes repressed by EC-8042 included important pluripotency factors like *SOX2* and *C-MYC*, markers of CSCs in different types of tumors, including sarcomas, like *ENG/CD105*, *ABCG2*, *KIT/CD117*, *CD44* and *ALCAM/CD166*, genes involved in proliferation and asymmetric division such as *LIN28B*, *ERBB2*, *SIRT1* and *KITLG*, genes involved in metastasis like *TWIST1*, *ZEB1* and *PLAT* and genes involved in relevant stemness signaling pathways like NOTCH signaling (*NOTCH1*, *MALML1* and *JAG1*), NFκB signaling (*NFκB1* and *IκBKB*) or Hippo signaling (*TAZ*, *WWC1*, *LATS1* and *YAP1*) (Figure 4C). On the other hand, Doxorubicin upregulated pluripotency genes such as *NANOG* and *POU5F1/OCT3/4*, CSC markers like *THY1/CD90*, *ALDH1A1*, *PROM1/CD133*, *KIT/CD117*, *CD24*, *CD38*, genes involved in metastasis like *TWIST1*, *TWIST2* and *ZEB2* and genes involved in signaling pathways like NOTCH signaling (*DLL1*, *DLL4* and *NOTCH2*), while also induced the downregulation of genes like *SOX2*, *ID1* or *ZEB1* (Figure 4D). IPA analysis of the group of genes differentially expressed after the treatments also showed that pluripotency signaling pathways were activated by doxorubicin but were inhibited by EC-8042 (Figure 4E). Western blotting analysis confirmed the repression of SOX2, C-MYC, NOTCH1, NFκB1 (p50 and p105) and SIRT1 protein synthesis by EC-8042 (Figure 4F).

These data are in line with previous works reporting that CSCs are resistant to doxorubicin treatment and therefore this drug may favor a CSC positive selection [32], and on the other hand, suggest that EC-8042 treatment may disadvantage the CSC phenotype in sarcomas.

### EC-8042 eliminates CSC subpopulations *in vitro*

**Figure 5:**
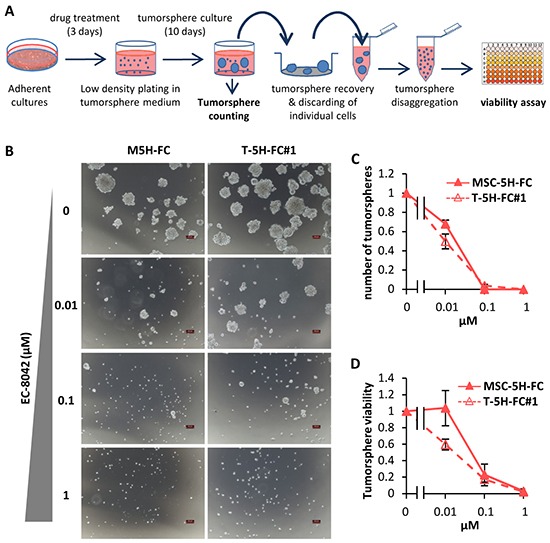
Effect of EC-8042 on tumorsphere-formation capacity **A.** Adherent cultures of sarcoma TICs were treated for 3 days with DMSO (carrier substance) or EC-8042. After wards that cells were plated at low density in tumorsphere medium and let to form tumorspheres for 10 days. Tumorspheres formed were scored, recovered, disaggregated and assayed for cell viability. **B.** Representative images of tumorspheres formed from MSC-5H-FC and T-5H-FC#1 cells treated with the indicated concentrations of EC-8042. Scale bars= 100μm. **C–D.** The effect of the drugs was estimated by scoring the number of tumorspheres formed relative to the untreated condition (C) or by measuring their cell viability (WST1 assay) **(D)** (n=3 independent experiments). Error bars represents the standard deviation.

**Figure 6:**
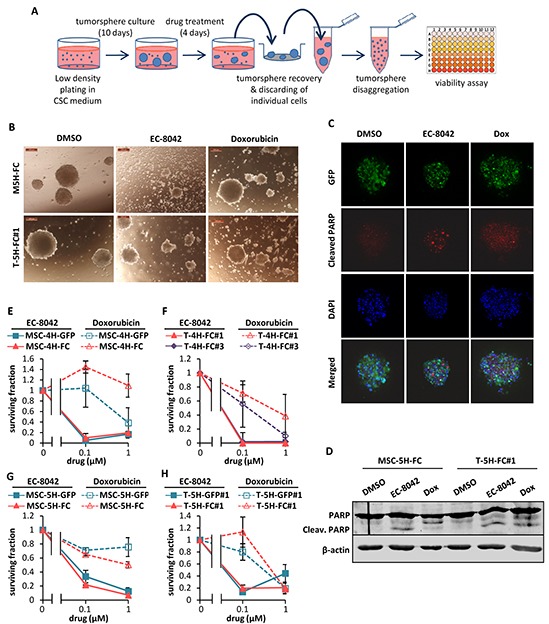
Effect of EC-8042 and doxorubicin on tumorsphere cultures **A.** Sarcoma TICs were plated at low density in CSC medium and let to form tumorspheres for 10 days followed by a 4-day treatment with DMSO (carrier substance), EC-8042 or doxorubicin. After treatment, the remaining tumorspheres were recovered, disaggregated and assayed for cell viability. **B.** Representative images of MSC-5H-GFP and MSC-5H-FC sarcosphere cultures treated with DMSO, 1 μM EC-8042 or 1 μM doxorubicin for 4 days. Scale bars= 200μm. **C.** Detection of cleaved PARP in GFP-positive T5H-FC#1 tumorspheres treated with DMSO, 0.5μM EC-8042 or 0.5μM doxorubicin for 48h. **D.** Western blot analysis showing the apoptotic cleavage of PARP in MSC-5H-FC and T-5H-FC#1 tumorsphere cultures treated with DMSO, 0.5μM EC-8042 or 0.5μM doxorubicin for the indicated times. **E–H.** Cell viability (WST1 assay) measured after the treatment of MSC-4H (E), T-4H (F), MSC-5H (G) and T-5H (H) tumorsphere cultures with the indicated concentrations of EC-8042 or doxorubicin. Error bars represents standard deviation.

To test whether EC-8042 may affect CSC subpopulations, we assayed the ability of the drugs to inhibit the formation of clonal spheres (tumorspheres) in nonadherent and serum free culture conditions (Figure 5A) [33]. EC-8042 efficiently reduced the number (Figure 5B–5C) and viability (Figure 5D) of the tumorespheres formed from MSC-5H-FC and T-5H-FC#1 cells in a dose-dependent manner. Likewise, EC-8042 treatment efficiently prevent tumorsphere formation from a primary patient-derived cell line (Supplementary Figure S3B). To further confirm the effect of EC-8042 on CSC subpopulations, we assayed cell viability after direct treatment of tumorsphere cultures (Figure 6A). After treatment with doxorubicin and especially with EC-8042, tumorspheres were smaller and displayed a disrupted and irregular morphology in all cell types (4H and 5H models) (Figure 6B & Supplementary Figure S5A–5B). Both drugs were able to induce apoptosis in tumorsphere cells as shown by PARP apoptotic cleavage induction both *in situ* (Figure 6C) or at protein level (Figure 6D). In any case, EC-8042 was much more efficient than doxorubicin in reducing the cell viability of tumorsphere cultures in all the studied sarcoma TICs (Figure 6E–6H). These results support that, in contrast to doxorubicin, EC-8042 could constitute an effective treatment to eliminate CSC subpopulations in sarcoma.

### EC-8042 inhibits the growth of MLS xenografts through the induction of a senescent-like state

**Figure 7:**
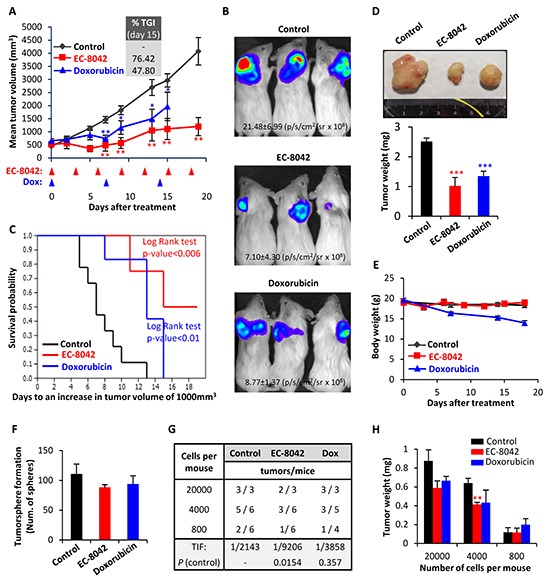
EC-8042 inhibits the growth of MLS sarcoma xenografts derived from transformed hMSCs Mice with established T-5H-FC#1 tumor xenografts were randomly assigned to 3 different groups (n=10, 6 and 5 in control, EC-8042 and doxorubicin groups respectively) and treated i.v. with saline buffer (control), EC-8042 at a dose of 18mg/Kg every 3 days (7 doses) or doxorubicin at a dose of 6 mg/Kg every 7 days (3 doses). **A.** Curves representing the mean tumor volume of T-5H-FC#1 xenografts during the treatments. Drug efficacy expressed as the percentage of tumor growth inhibition (%TGI) on day 15 is indicated. **B.** *In vivo* bioluminescence of tumors generated from luciferase-expressing T5H-FC#1 cells in a 3 mice-cohort of the indicated series at day 8 after the beginning of the indicated treatments. Average radiance values ± standard deviation are presented. **C.** Kaplan-Meier curves generated using the reaching of a tumor volume of 1000 mm^3^ as end-point event. **D.** Representative images (upper panel) and tumor weight (bottom panel) at the end of the experiment. **E.** Mean body weight of mice during the treatments. Doxorubicin was toxic and caused weight loss during the treatment. **F–G.** For the evaluation of CSC subpopulations after drug treatments we harvested xenograft tumors (n=3 per group) after a single dose treatment of EC-8042 (50mg/Kg) or doxorubicin (8 mg/kg) and dissociated them into single cells to evaluate the number of tumorspheres formed (F) and the tumor re-initiation ability in limiting dilution assays (G). The number of mice that grew tumors at week 3 and total number of inoculated mice for each condition is indicated (G). TIF was calculated using ELDA software. **H.** Mean tumor weight for each group at the end of the experiment (week 5). Error bars represents the standard error of the mean (SEM) and asterisks indicate a statistically significant difference in tumor volumes between the EC-8042 or doxorubicin-treated and control groups (*:p<0.05, **:p<0.005, ***: p<0.0005; two-sided Student *t* test). The log-rank test p value was used to estimate significant differences between control and drug treated groups in Kaplan-Meier analysis. In limiting dilutions assays Pr (>chiSq) referring to the control tumors are indicated.

EC-8042 treatment induced a profound tumor growth inhibition in MLS xenografts originated from T-5H-FC#1 cells (Figure 7 & Supplementary Figure S6). A reduction in tumor growth was observed early during the treatment and highly significant differences in tumor volume and luminiscence levels between control and EC-8042-treated tumors were observed as soon as 5-8 days after the beginning of the treatment (Figure 7A–7B). At the end-point, two different experiments displayed TGI percentages of 61.4 and 76.42% respectively, and showed highly significant differences in survival (Figure 7A & 7C and Supplementary Figure S6). Likewise, tumor weights in the control series almost triplicated those of the EC-8042-treated series at the end of the experiment (Figure 7D). On the other hand, doxorubicin treatment showed lower antitumor activity compared to EC-8042 and resulted toxic at the later time points (Figure 7E). To examine the ability of drugs to target CSC subpopulations *in vivo*, we harvested xenograft tumors after a single high dose (50 mg/Kg) treatment of EC-8042 or doxorubicin and dissociated them into single cells to evaluate their self-renewal and tumor re-initiation ability. A non-significant reduction in tumorshpere formation was observed in EC-8042-treated tumors (Figure 7F). More importantly, EC-8042 produced a delay in tumor formation after re-implantation of tumor cells in limiting dilution assays. We initially (week 3) observed a 4-fold significant decrease of TIF in EC-8042- *versus* control-treated tumors (Figure 7G). Although eventually (week 5) tumor growth was detected in nearly all mice (13 out of 15 in control and EC-8042 groups and 12 out of 12 in doxorubicin group), tumor weights in the EC-8042 group were significantly lower than in control group (Figure 7H). These data suggest that EC-8042 is able to partially reduce CSCs subpopulations *in vivo*.

Intra-tumor pharmacokinetic measurement of EC-8042 concentration at 24h and 48h after 50 mg/kg single dose treatment indicated that the drug accumulated at doses higher that those deemed active *in vitro:* 1640 ± 105 ng/g (media ± standard deviation) at 24h and 412 ± 267 ng/g at 48h. This high dose treatment of EC-8042 is below the single maximum tolerated dose [17] and was able to produce tumor regression in a lung cancer xenograft model (unpublished data).

**Figure 8:**
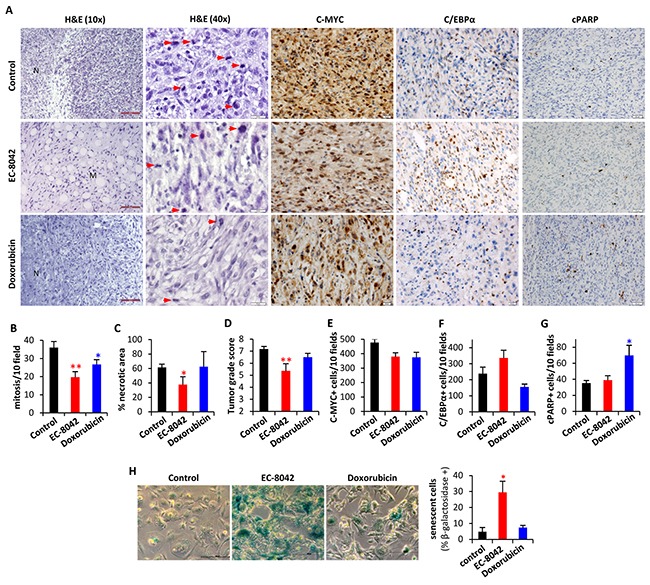
*In vivo* anti-tumor activity of EC-8042 is associated to the induction of a senescent-like state Pathological analysis of T5H-FC#1-generated tumors treated i.v. with saline buffer (control), EC-8042 at a dose of 18mg/Kg every 3 days (7 doses) or doxorubicin at a dose of 6 mg/Kg every 7 days (3 doses). **A.** H&E staining and immuno-staining detection of C-MYC, C/EBPα and cleaved PARP (cPARP). Necrotic (N) and myxoid (M) areas, and mitotic cells (red arrows) are indicated. Scale bars= 100μm (H&E 10X); 50μm (C-MYC, C/EBPα and c-PARP); and 20 μm (H&E 40X). **B–G.** Quantification of tumor-related features including mitosis [number of mitotic figures per 10 high power fields (40X)] (B), necrosis (C), tumor grade score according to FNCLCC system (D), C-MYC (E) and C/EBPα (F) nuclear expression [number of cell showing nuclear staining per 10 high power fields (40X)] and apoptotic cells [number of cell showing nuclear staining for c-PARP and displaying apoptotic morphology per 10 high power fields (40X)] (G) in tumors from the indicated series. **H.** Analysis of senescent-associated β-galactosidase activity in *ex vivo*-established cell lines derived from tumors of the indicated series (n=4). Representative images (left panels; scale bars= 20 μm) and the quantification of the percentage of β-galactosidase + cells in 10 randomly selected fields (>600 total cells) of each cell line (right panel) are presented. Error bars represents the SEM and asterisks indicate a statistically significant difference in tumor volumes between the EC-8042 or doxorubicin-treated and control groups (*:p<0.05, **:p<0.005; two-sided Student *t* test).

Histological examination of tumors showed that, in comparison with control tumors, EC-8042-treated tumors presented more extended areas of myxoid pattern and increased adipogenic differentiation (Figure 8A). In addition, both doxorubicin and especially EC-8042 treated tumors presented significantly lower mitotic counts than untreated tumor samples (Figure 8A–8B). Moreover, all of the tumors of the control and doxorubicin series displayed a necrotic central area associated to their aggressive growth pattern, and the appearance of this necrotic area is largely prevented by EC-8042 treatment (Figure 8A & 8C). Therefore, tumors treated with EC-8042 showed a significant reduction in tumor grade according to the score obtained by applying the three-grade FNCLCC system which depend on the necrosis, mitotic counts and differentiation status of the tumors (Figure 8D). Immunohistochemical analysis confirmed a decrease of C-MYC nuclear and cytosolic expression in EC-8042 treated tumors which did not reach statistical significance (Figure 8A & 8E). Likewise, in line with the observed increase in adipogenic differentiation, EC-8042 produced an increase in the expression of C/EBPα (Figure 8A & 8F), which is in any case much more modest than that observed in TICs cultures. In addition, only doxorubicin treatment induced a significant increment in apoptotic cell death (cleaved-PARP staining) (Figure 8A & 8G). The fact that EC-8042-treated tumors showed a great reduction in the mitotic index suggest that this drug could induce a senescent-like state associated to a permanent cell-cycle arrest, as previously observed for other anti-tumor drugs [34, 35]. To test this possibility, we disaggregated and placed-back in culture four different tumors from each group and we analyze senescence-associated β-galactosidase in these *ex vivo*-established cell lines. Cultures derived from EC-8042-treated tumors showed a pronounced increase in size characteristic of senescent cells and a large and significant increase in the fraction of β-galactosidase positive cells (Figure 8H). Furthermore, immunohistochemical analysis showed a highly increased expression of P16-INK4A, a key regulator of cellular senescence, in EC-8042-treated tumors (Supplementary Figure S7). Taken together, these results indicate that the *in vivo* sarcoma growth inhibition induced by EC-8042 is due to the induction of a senescent-state, while the anti-tumor effects of doxorubicin are mainly caused by the promotion of apoptotic cell death.

## DISCUSSION

In this work, we aimed to find new targeted therapies against soft tissue sarcomas using a model of sarcomagenesis in which, UPS or MLS arise from the cell-of-origin targeted with oncogenic mutations relevant for sarcoma development [3, 25, 36]. In search for altered transcription factor-mediated signaling, we found that SP1 is the most significantly altered transcription regulator in this model. Notably, a pro-tumorigenic role for SP TFs has been reported in several types of soft tissue sarcomas. Thus, SP1, SP3 and SP4 have been found overexpressed in rhabdomyosarcoma cells and fibrosarcoma-forming transformed human fibroblasts, and its downregulation using tolfenamic acid, reactive oxygen species (ROS)-inducing anticancer agents or specific siRNAs/U1snRNAs resulted in inhibited tumor growth [6, 8, 37, 38]. To inhibit the activity of SP1 and other SP family members in sarcoma TICs we have used a newly developed mithralog which shows a potent anti-tumor activity while being less toxic than MTM and other mithralogs [17]. EC-8042 induced strong anti-proliferative effects that can be reproduced by SP1 knockdown using RNA interference. This data suggest that the inhibition of this transcription factor is a relevant mechanism of action for EC-8042, as previously demonstrated for some ROS-inducing drugs [38].

Importantly, we proved that this compound, EC-8042, is not recognized as a substrate by transporters of the ABC family. Several studies reported that SP1 and SP3 regulated the expression of ABC family member genes in different types of cancer cells, and that inhibition of SP1 activity with MTM downregulated the expression of ABC family genes and CSC-associated genes, inhibited proliferation and tumorigenicity, suppressed CSC-associated properties and caused chemosensitization to anti-cancer drugs [28, 29, 39–41]. Similarly, we show that SP1 inhibition using EC-8042 results in the downregulation of *ABCG2*, *ABCB1* and *ABCC1*. In addition, EC-8042, opposite to doxorubicin, repressed the expression of many genes involved in the development of the CSC phenotype.

The amplification of *C-MYC*, one of the oncogenic genes with enhanced expression in our model of sarcomagenesis, is a frequent event in many types of sarcoma, including MLS [42]. EC-8042 treatment downregulates the expression of this oncogene in our model, in line with that described in ovarian cancer cells [20]. C-MYC may be regulated independently of SP1 by several anti-cancer drugs [38]. We showed that siRNA-mediated knockdown of SP1 induced C-MYC downregulation, suggesting that EC-8042 effect on C-MYC expression is mediated by SP1. In addition, genes involved in NOTCH signaling display a striking differential regulation after EC-8042 and doxorubicin treatments. In a similar way, the expression of the Hedgehog signaling receptor *PTCH1* is repressed by EC-8042 but stimulated by doxorubicin. Importantly, both NOCHT and Hedgehog signaling routes have been found activated in CSC subpopulations of UPS, and their inhibition reduced the proportion of cells displaying CSC properties and suppressed tumor self-renewal [43].

The anti-tumor effect of EC-8042 in this model of sarcomagenesis was associated to a stronger reduction of the mitotic index, the induction of adipogenic differentiation, the initiation of a senescent-like state and the ability to target CSCs. Notably, senescence bypass in MSCs have been suggested as plausible mechanism for sarcoma development and therefore, senescence induction therapy could represent an efficient strategy against sarcoma [44]. Altogether, these data suggest that EC-8042 could act as an anti-pluripotency therapy able to target both CSCs and non-CSCs populations derived from sarcoma TICs.

## MATERIALS AND METHODS

### Cell types and drugs

Transformed hBMSCs cell lines were previously generated and characterized (Supplementary Figure S1A; [23–25]. The identity of transformed hBMSCs has been authenticated by Short Tandem Repeats analysis during the last 3 months. All the cell types were cultured as previously described [45]. Luciferase-expressing T-5H-FC#1 cells were generated as described in Supplemental Information. Gene expression profiling of some of these cell types was previously reported and deposited (GSE48030; [25]). Analysis of upstream transcription regulator signaling significantly altered in transformed hBMSCs as compared to wt hBMSCs was performed with the list of differentially expressed genes (p value <0.05; regulation ≤2-fold) using Ingenuity Pathway Analysis (IPA) software 8.0 (Ingenuity Systems, Inc., Redwood City, CA). Stocks of EC-8042 and Doxorubicin (Sigma, St Louis, MO) were prepared as 1 mM solutions in sterile DMSO for *in vitro* experiments, maintained at −20°C, and brought to the final concentration just before use. For *in vivo* experiments, EC-8042 and doxorubicin were prepared in sterile saline solution. All experimental protocols have been performed in accordance to approved institutional review board guidelines and were approved by the Institutional Ethics Committee of the Hospital Universitario Central de Asturias.

### RT-qPCR assays

The expression of *SP1*, *C-MYC*, *XIAP*, *ABCB1*, *ABCG2* and *ABCC1* was assessed by qPCR as described in Supplemental Information. Primer sequences used are shown in Supplementary Table S1.

The Human Cancer Stem Cells RT2 Profiler PCR Array (PAHS-176-Z; SABiosciences, Qiagen Iberia, Madrid, Spain) was used to analyze the expression of 84 genes linked to CSCs properties according to the manufacturer instructions (Supplemental Information; Supplementary Table S2). The analysis of altered signaling associated to genes affected by EC-8042 was performed using Ingenuity Pathway Analysis (IPA) software 8.0 (Ingenuity Systems, Inc., Redwood City, CA).

### Western blot

Whole cell protein extraction and western blot analysis were done as previously described [45]. Antibodies used are described in Supplemental Information.

### siRNA transfection

SP1-especific siRNAs were from Sigma (siSP1#1; HU-01823-1) and from Dharmacon (Lafayette, CO) (siSP1#2; J-026959-05-0002). siGenome RiSC-Free control siRNA (siControl; D-001220-01) were from Dharmacon. siRNA sequences are shown in Supplementary Table S1. Cells were transfected with 100 pmol/ml siRNAs using Lipofectamine 3000 (Life Technologies) according to the manufacturer's instructions. 48 or 72 hours after transfection, cells were collected and analyzed [46].

### Transporter substrate and inhibition assays

The assessment of the ability of EC-8042 and MTM to inhibit ACBB1/MDR1 and ABCG2/BCRP1 or to be a substrate for these transporters were performed by Absorption Systems (Exton, PA) using MDR1- or BCRP-expressing MDCK cells as described in Supplemental Information.

### Cell viability assays

The viability of all cell lines in the presence and absence of drugs was determined using the Cell Proliferation reagent WST-1 (Roche, Mannheim, Germany) (Supplemental Information).

### Tumorsphere culture

Cells lines or dissociated xenografts were plated at a density of 5,000 cells per well in 6-well plates treated with a sterile solution of poly 2-hydroxyethyl methacrylate (10 g/l in 95% ethanol; Sigma) to prevent cell attachment, in serum-free sphere medium containing DMEM-F12 (GE Healthcare, Pittsburg, PA) supplemented with Glutamax (1:100; Life Technologies), B-27 Supplement (1:50; Life Technologies), Heparin (1:1000; Sigma), the growth factors human EGF (20 ng/ml) and human bFGF (10 ng/ml; PeproTech, London, UK) and 1% methylcellulose (Sigma) to avoid cell aggregation. In addition, fresh aliquots of EGF and bFGF were added every three days. In xenograft-derived experiments, well-rounded spheres formed after 10 days of culture were counted. To analyze the effects of drugs *in vitro*, we treated adherent cultures with increasing concentrations of drugs for 72 h and then we grew them in tumorsphere culture conditions to assay the ability of the drugs to inhibit the formation of tumorespheres. Alternatively, 10-days spheres were incubated in sphere medium containing different concentrations of EC-8042 and doxorubicin for 4 days. After that, tumorspheres were scored, recovered by filtration through 70-μm cell strainers (Corning, New York, NY), washed with PBS and disaggregated by incubation with trypsin (0.25%)/EDTA (Life Technologies) for 15 min. The resulting cell suspension was pelleted and resuspended in 1ml of normal growth medium containing 10% FBS (Life Technologies). 150 μl of these cells suspensions were plated in triplicates in 96 well plates and incubated overnight to allow their attachment. Finally, cell viability was determined using the Cell Proliferation reagent WST-1 as above.

### Immunofluorescence staining

Tumorspheres cultures were washed with phosphate-buffered saline (PBS) twice and fixed in 4% paraformaldehyde (Sigma) in PBS for 20 minutes. Tumorspheres were then washed 3 times with PBS, permeabilized in PBS plus 0.1 % triton X-100 (Sigma) for 20 minutes, washed another 5 times with PBS and incubated with the blocking solution (10% goat serum in PBS) for 1 hour. After that, tumorspheres were incubated with anti-cleaved PARP [(ab32064), 1:500 dilution] from Abcam (Cambridge, UK) for 24 hours, washed 4 times with PBS plus 0.1% Tween-20 for 10 minutes, incubated with Alexa-555-conjugated anti-rabbit IgG [(A-21422), 1:300 dilution] from Life Technologies for 1 hour, washed another 4 times with PBS plus 0.1% tween at 4 ºC and finally incubated with 300 ng/ml DAPI (Sigma) in PBS for 24 hours at 4 ºC. The slides were washed extensively with PBS and pipetted into a drop of ProLong® Gold Antifade Mountant medium (P36930) from Life Technologies deposited in μ-Slide 8-well chambered coverslips (80826) from Ibidi (Planegg, Germany). Confocal sections images obtained with identical exposure times were collected using an Ultra-Spectral TCS-SP2-AOBS confocal microscope (Leica, Wetzlar, Germany).

### Cell-cycle analysis

Cell-cycle analysis of floating and adherent cells was carried out as described previously [46].

### Senescence-associated β-galactosidase staining

Cells were fixed and incubated overnight with X-gal solution (pH 6.0) as previously described [35].

### Xenograft experiments

Female NOD/SCID mice of 5-6 weeks old (Janvier Labs, St Berthevin, France) were inoculated subcutaneously (s.c.) with 1×10^6^ T5H-FC#1 cells. Once tumors reached 200-500 mm^3^, the mice were randomly assigned to receive intra-venous (i.v.) treatments of saline solution (control), EC-8042 at a dose of 18 mg/kg every 3 days or doxorubicin at 6 mg/kg every 7 days. Animals were sacrificed by CO_2_ asphyxiation when the tumors reached approximately 2 cm. Mean tumor volume differences between groups were determined using a caliper or measuring the luminescence intensity using an IVIS Spectrum (Caliper Life Sciences, Hopkinton, MA) (Supplemental Information). The student *t* test was performed to determine the statistical significance between control and treated groups. Survival was represented using Kaplan-Meier analysis and the log-rank test to estimate significant differences among groups (PAST 3.01 software). Drug efficacy was expressed as the percentage tumor growth inhibition (%TGI) (Supplemental Information). Upon tumor removal, a portion of some of the tumors was mechanically disaggregated to establish *ex-vivo* tumor cell lines as described [47]. In experiments aimed to analyze CSCs subpopulations after treatments, a single dose of EC-8042 (50 mg/kg) or doxorubicin (8 mg/kg) were inoculated 48 hours before tumor extraction. Tumors were dissociated into single cell suspensions using MACS Tissue Dissociation Kit and the GentleMACS Dissociator system (Miltenyi Biotec, Bergisch Gladbach, Germany). Mouse cells were removed from cell suspensions using Mouse Cell Depletion Kit (Miltenyi Biotec). For limiting dilution assays, serially diluted numbers of viable cells dissociated from xenografts were injected s.c. Tumor growth initiation was monitored for 5 weeks and tumor-initiating frecuency (TIF) was calculated using the ELDA software [48]. Drug accumulation in tumors was analyzed as described in Supplemental Information. All animal research protocols were approved by the Animal Research Ethical Committee of the University of Oviedo prior to the study.

### Histological analysis

Tumor samples were fixed in formol, embedded in paraffin, cut into 4-μm sections, and stained with hematoxylin and eosin (H&E). Tumor sections were also subjected to immunohistochemistry using the antibodies described in Supplemental Information [49]. Tumor grade was analyzed in H&E stained preparations using the French Federation of Comprehensive Cancer Centers (FNCLCC) grading system (Supplemental Information).

## SUPPLEMENTARY FIGURES AND TABLES




