# Supplementary material for: Inhibition of SP1 by the mithramycin analog EC-8042 efficiently targets tumor initiating cells in sarcoma
Source: Oncotarget. 2016 Apr 18;7(21):30935–50. doi: 10.18632/oncotarget.8817 (PMC5058729; doi:10.18632/oncotarget.8817)
Supplement: Supplementary file 1 [file oncotarget-07-30935-s001.pdf]

Inhibition of SP1 by the mithramycin analog EC-8042 efficiently targets tumor initiating cells in sarcoma

SUPPLEMENTARY FIGURES AND TABLES

A

BM-hMSC	Hit#1	Hit#2	Hit#3	Hit#4	Hit#5	Hit#6	Oncogenic status	Tumor type
BMSC	-	-	-	-	-	-	wild type	-
hMSC-4H-GFP	ectopic expression of hTERT	inactivation of P53 by expression of E6 antigen of the HPV16	inactivation of RB by expression of E7 antigen of the HPV16	introduction of SV40-ST to inactivate PPA2 leading to C-MYC stabilization	-	GFP	immortalized	-
hMSC-4H-FC						FUS-CHOP	transformed	myxoid liposarcoma
T-4H-FC#1						FUS-CHOP	xenograft-derived line	myxoid liposarcoma
T-4H-FC#3						FUS-CHOP	xenograft-derived line	myxoid liposarcoma
hMSC-5H-GFP					expression of oncogenic H-RAS^{v-12}	GFP	transformed	Undiff. sarcoma
hMSC-5H-FC						FUS-CHOP	transformed	myxoid liposarcoma
T-5H-GFP#1						GFP	xenograft-derived line	Undiff. sarcoma
T-5H-FC#1						FUS-CHOP	xenograft-derived line	myxoid liposarcoma

B

MSC-5H-GFP vs BMSC

Transcription Regulator Signaling	Activation z-score	p-value of overlap
TP53	-4.934	3.46E-32
NUPR1	-5.01	5.22E-23
SP1	1.575	1.56E-22
RB1	-3.422	7.25E-21
NFKBIA	0.065	1.92E-19
STAT3	1.112	4.68E-19
E2F4	-0.478	1.80E-18
CEBPB	0.932	9.95E-18
YY1	-0.611	1.38E-17
CTNNB1	0.856	1.96E-17
SMARCA4	-2.16	6.13E-17
CEBPA	-0.724	1.32E-16
JUN	1.867	2.05E-16
RELA	2.785	1.82E-15
CDKN2A	-5.566	5.75E-15
SMARCB1	-3.06	3.14E-13
TBX2	4.292	9.30E-13
FOS	0.922	1.11E-12
CREB1	0.986	6.68E-12
EZH2	2.254	7.24E-12
NFKB1	1.479	8.29E-12
E2F1	3.505	1.38E-11
FOXO1	1.301	1.65E-11
FOXM1	4.029	2.02E-11
NEUROG1	-0.577	1.16E-10

MSC-5H-FC vs BMSC

Transcription Regulator Signaling	Activation z-score	p-value of overlap
TP53	-5.053	4.91E-30
NUPR1	-5.793	2.96E-24
STAT3	0.86	1.80E-23
SP1	1.641	8.06E-22
RB1	-3.195	2.34E-20
E2F4	-0.113	2.88E-19
SMARCA4	-1.962	4.27E-19
NFKBIA	1.282	2.35E-18
CEBPB	-0.008	3.68E-18
YY1	0.446	1.02E-17
CEBPA	-1.179	1.59E-17
CTNNB1	0.02	8.71E-17
CDKN2A	-5.84	3.69E-15
TBX2	4.608	4.93E-15
FOXM1	4.047	5.03E-15
RELA	3.113	5.51E-15
E2F1	4.118	1.50E-14
JUN	2.742	1.54E-14
SMARCB1	-2.879	3.96E-14
STAT1	2.268	1.13E-13
EZH2	2.623	1.38E-13
NFKB1	1.571	1.51E-13
FOS	1.94	3.52E-12
FOXO1	1.286	5.28E-12
E2F3	1.608	6.18E-12

T-5H-FC#1 vs BMSC

Transcription Regulator Signaling	Activation z-score	p-value of overlap
TP53	-4.645	7.60E-39
STAT3	1.023	1.35E-28
NFKBIA	1.176	5.38E-23
NKX2-3	-2.368	1.98E-21
NUPR1	-4.73	7.99E-21
CTNNB1	0.613	2.07E-20
SP1	1.437	1.75E-19
IRF7	6.552	3.19E-18
STAT1	4.085	7.42E-18
RELA	3.945	1.76E-17
SMARCA4	-1.518	3.65E-17
YY1	0.483	4.62E-17
RB1	-3.853	9.62E-17
CEBPA	-0.124	1.54E-16
E2F4	-0.426	2.84E-16
JUN	1.72	8.17E-16
HDAC1	-1.782	2.21E-15
SMARCB1	-2.127	7.60E-14
E2F1	3.476	1.11E-13
TBX2	4.175	1.54E-13
CEBPB	0.739	4.13E-13
EZH2	2.746	4.14E-13
PPRC1	3.859	7.58E-13
NFKB1	2.52	1.44E-12
IRF1	4.161	3.24E-12

Supplementary Figure S1: SP1-mediated signaling is altered in sarcoma-initiating transformed hBMSCs. A. Summary of the main features (transforming hits and oncogenic status) of the hBMSC-derived types used in this study. **B.** List of transcription factor-mediated signaling pathways most significantly altered in MSC-5H-GFP (left), MSC-5H-FC (middle) or T5H-FC#1 (right), as compared to wild type hBMSCs, obtained using the IPA software. SP1 data are highlighted.

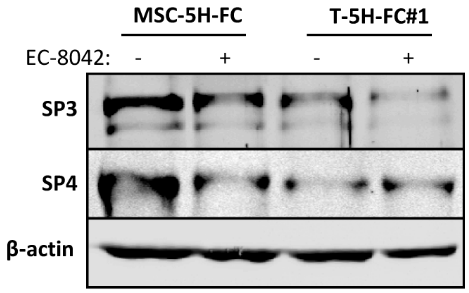

Supplementary Figure S2: Protein levels of SP3, SP4 and β-actin in MSC5H-FC and T5H-5H-FC#1 cells treated with 0.5µM EC-8042 for 24 hours. MSC-5H-FC cells show higher levels of SP3 and SP4 than T-5H-FC#1 and EC-8042 treatment decreases SP3 expression in both cell types and SP4 in MSC-5H-FC.

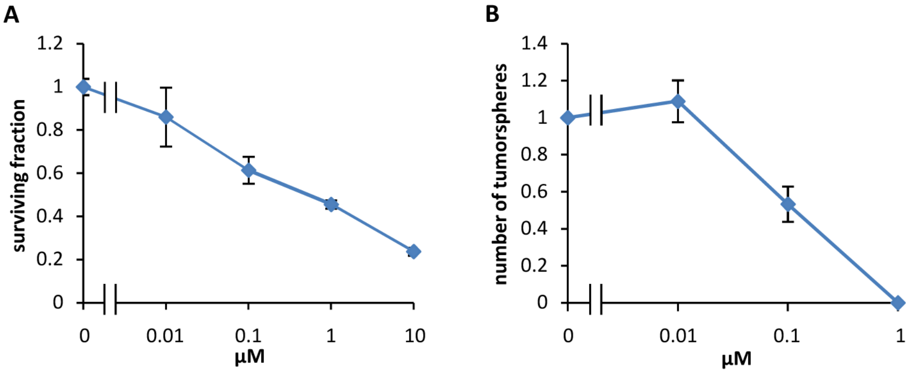

Supplementary Figure S3: Effect of EC-8042 on a patient-derived cell line. A. Cell viability (WST1 assay) measured after the treatment of a primary cell line (CDS10+) derived from a chondrosarcoma patient with increasing concentrations of EC-8042 for 72 hours. IC_{50} value is 0.457 µM. **B.** Analysis of the effect of drug on the ability to form tumorspheres. Adherent cultures of the primary cell line CDS10+ were treated for 3 days with increasing concentrations of EC-8042. After that cells were plated at low density in tumorsphere medium and let to form tumorspheres for 10 days. The effect of the drugs was estimated by scoring the number of tumorspheres formed relative to the untreated condition. Error bars represents the standard deviation (n=3 independent experiments).

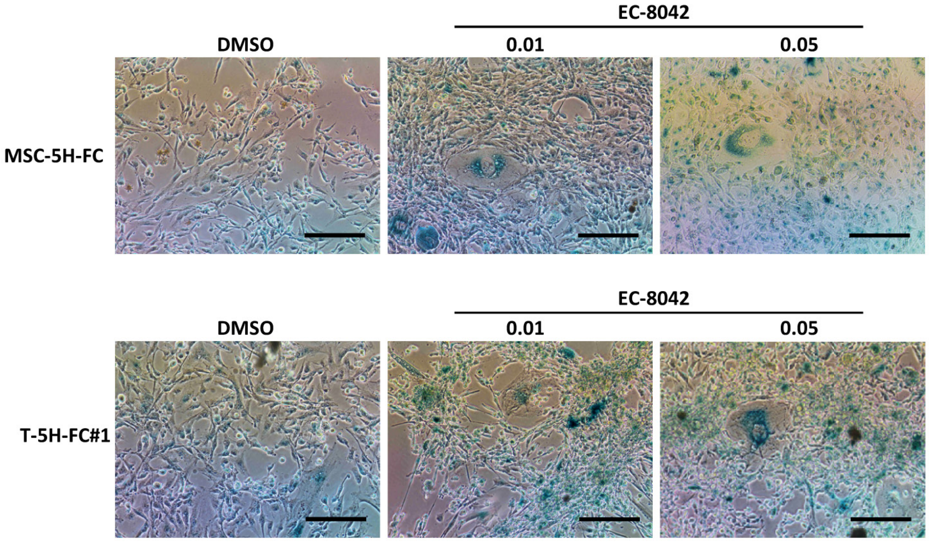

Supplementary Figure S4: Senescence-associated β-galactosidase staining in adherent cultures of MSC-5H-FC and T-5H-FC#1 cells treated for 96h with DMSO or the indicated concentrations of EC-8042. Representative images of 3 independent experiments. Scale bars= 200µm.

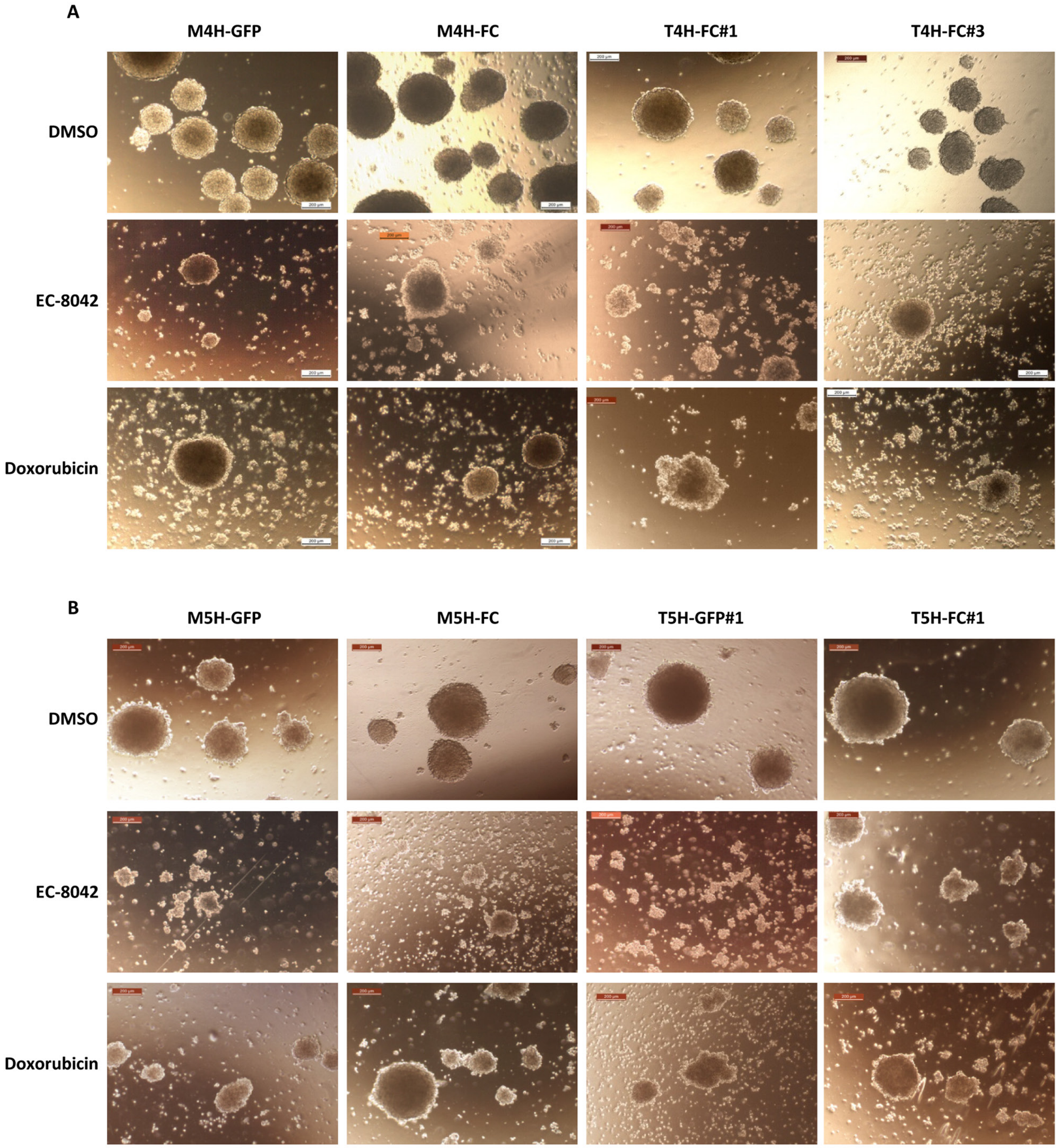

Supplementary Figure S5: A-B. Images representative of MSC-4H / T-4H (**A**) and MSC-5H / T-5H **(B)** cell types-derived tumoresphere cultures treated with carrier substance (DMSO), 1 μM EC-8042 or 1 μM doxorubicin.Scale bars= 200μm.

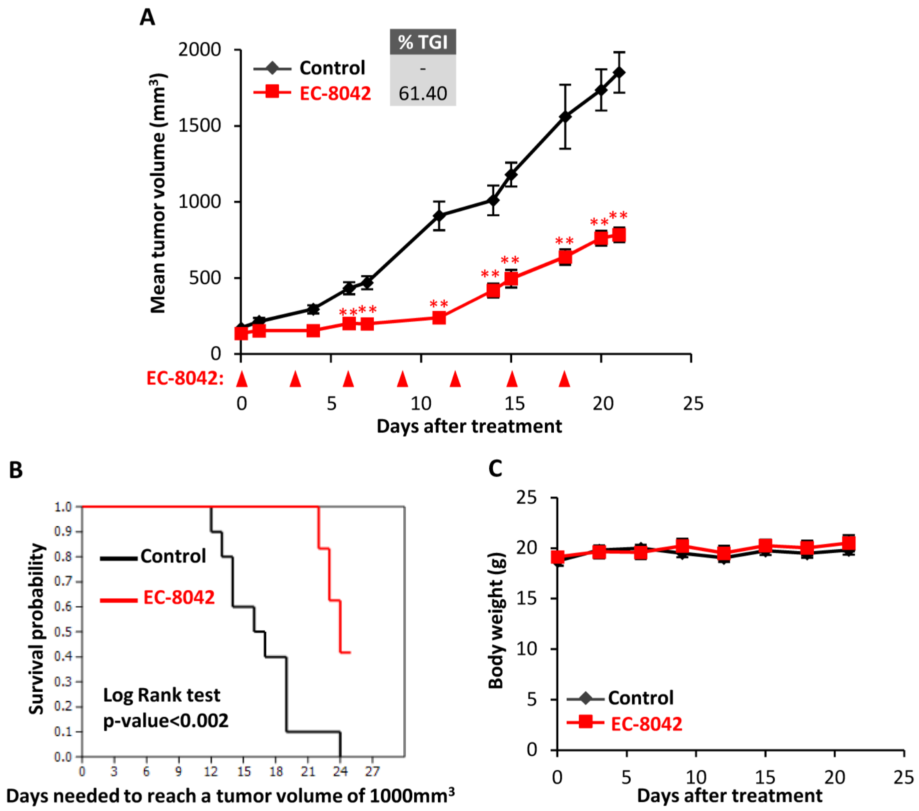

Supplementary Figure S6: Effect of EC-8042 on T-5H-FC#1 tumor xenografts growth. Mice with established tumors were randomly assigned to 3 different groups (n=10 per group) and treated i.v. with saline solution (control) or EC-8042 at a dose of 18mg/Kg every 3 days (7 doses). **A.** Curves representing the mean tumor volume of T-5H-FC#1 xenografts during the treatments. Drug efficacy expressed as the percentage of tumor growth inhibition (%TGI) at the end of the experiments is indicated. **B.** Kaplan-Meier curves using the reaching of a tumor volume of 1000 as end-point event. **C.** Mean mice body weight during the treatment periods. EC-8042 treatment was not toxic and did not cause weight loss. Error bars represents the standard error of the mean (SEM) and asterisks indicate a statistically significant difference in tumor volumes between the EC-8042-treated and control groups (*:$p<0.05$, **:$p<0.005$; two-sided Student *t* test). The log-rank test p value was used to estimate significant differences between control and drug treated groups in Kaplan-Meier analysis.

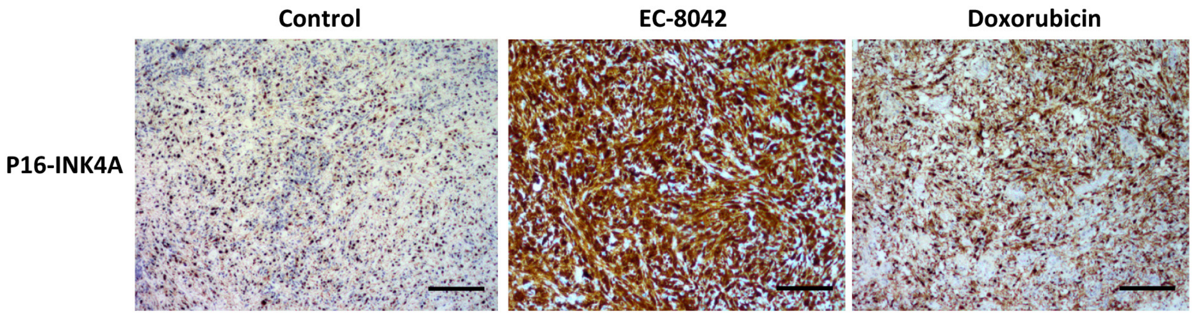

Supplementary Figure S7: Imuno-staining detection of P16-INK4A in T5H-FC#1-generated tumors treated i.v. with saline buffer (control), EC-8042 at a dose of 18mg/Kg every 3 days (7 doses) or doxorubicin at a dose of 6 mg/Kg every 7 days (3 doses). Scale bars= 100µm.

Supplementary Table S1: Sequences of primers and siRNAs used in the present study

Primers		
**Gene**	**Forward (5′ → 3′)**	**Reverse (5′ → 3′)**
**ABCB1**	GTGGTGGGAACTTTGGCTG	TACCTGGTCATGTCTTCCTCC
**ABCC1**	ATGTCACGTGGAATACCAGC	GAAGACTGAACTCCCTTCCT
**ABCG2**	ATGGATTTACGGCTTTGCAG	TCTTCGCCAGTACATGTTGC
**C-MYC**	TGCTCCATGAGGAGACACC	CTTTTCCACAGAAACAACATCG
**SP1**	CTATAGCAAATGCCCCAGGT	TCTGGGCTGTTTTCTCCTTC
**XIAP**	TTTTGGGACATGGATATACTCAGTT	AGCACTTTACTTTATCACCTTCACC
**β-Actin**	CGTCTTCCCCTCCATCG	CTCGTTAATGTCACGCAC
**siRNAs**		
**siRNA**	**Reference**	**Sequence**
**siSP1#1**	esiRNA HU-01823-1 (Sigma)	CCGCTCCCAACTTACAGAACCAGCAAGTT<br>CTGACAGGACTACCTGGAGTGATGCCTAA
**siSP1#2**	On-Target plus siRNA J-026959-05-0002 (Dharmacon)	GCCAAUAGCUACUCAACUA

Supplementary Table S2: Cancer Stem Cell RT-PCR array.

See Supplementary File 1
